# Necroptosis induced by MLKL overexpression in liver triggers cellular senescence and leads to chronic inflammation and fibrosis

**DOI:** 10.1007/s11357-025-01994-y

**Published:** 2025-11-24

**Authors:** Ramasamy Selvarani, Sunho Lee, Mani Saminathan, Puvarajan Boovalingam, Kavitha Kurup, Kevin Pham, Roman F. Wolf, Willard M. Freeman, Archana Unnikrishnan, Arlan Richardson

**Affiliations:** 1 Biochemistry & Physiology, University of Oklahoma Health Sciences, Oklahoma City, OK USA; 2 ICAR-Indian Veterinary Research Institute, Izatnagar, Bareilly, Uttar Pradesh India; 3 Regional Research and Educational Centre, TANUVAS, Pudukkottai, Tamilnadu India; 4 Genes & Human Disease Program, Oklahoma Medical Research Foundation, Oklahoma City, OK USA; 5 Oklahoma City Veterans Affairs Health Care System, Oklahoma City, OK USA; 6 Harold Hamm Diabetes Center, OU Health, Oklahoma City, OK USA; 7 Oklahoma Center for Geroscience and Healthy Brain Aging, Oklahoma City, OK USA

**Keywords:** Necroptosis, Cellular senescence, Chronic inflammation, Inflammaging, Fibrosis, MLKL

## Abstract

Cellular senescence and necroptosis are two cell fates, which trigger an inflammatory response and increase with age, that have been proposed to play a role in inflammaging. In this study, we performed the first study to directly test the possible interaction between necroptosis and cellular senescence. Using a novel *Mlkl*-KI mouse model, we were able to specifically induce (~ 4-fold) the overexpression of MLKL, the necroptotic executioner, in hepatocytes (h*Mlkl*-KI mice). The overexpression of MLKL led to increased necroptosis and cell damage/death in liver, as shown by increased levels of MLKL-oligomers, TUNEL staining, and Ki-67 staining in the livers, as well as increased ALT activity and HMGB1 levels in the plasma. The increase in necroptosis was paralleled by an increase in cellular senescence. We observed increased levels of *p16*^INK4A^ and *p21*^Clip1/Waf1^ as well as SASP-factors. As expected, inflammation, as measured by the levels of proinflammatory factors and mononuclear cell clusters, was increased in the h*Mlkl*-KI mice. Transcriptomic analysis revealed that MLKL overexpression altered the expression of genes involved in cellular senescence, inflammation, and drug metabolism. The increase in inflammation, necroptosis, and cellular senescence in the livers of 6-month-old h*Mlkl*-KI mice was associated with an increase in liver fibrosis. The data from our study suggest that necroptosis has the potential of inducing inflammation through two pathways: (1) the initial inflammatory storm triggered by DAMPs released from necroptotic, dying cells and (2) SASP-factors produced by senescent cells that were induced by necroptosis.

**Graphical abstract:**

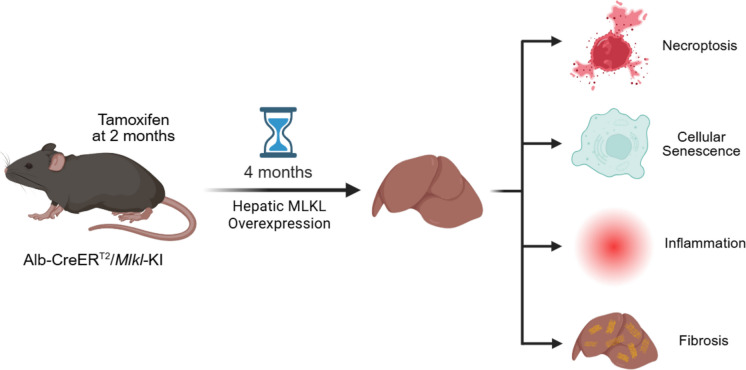

**Supplementary Information:**

The online version contains supplementary material available at 10.1007/s11357-025-01994-y.

## Introduction

Inflammation is a double-edged sword, depending on whether inflammation exposure is acute or remains prolonged. Chronic inflammation, termed inflammaging, is one of the hallmarks of aging and contributes to tissue damage as well as the pathogenesis of degenerative diseases [1, 2]. Overwhelming evidence has shown that serum levels of proinflammatory cytokines, such as IL-1β, IL-6, and TNF-α, increase with age in old animals and humans, which are associated with an increase in various age-related diseases and frailty [3]. Thus, inflammaging is recognized as one of the major risk factors for both morbidity and mortality in older adults [4]. Based on the analysis conducted by Global Burden of Disease (GBD), more than 50% of all deaths are due to inflammatory diseases [3, 5]. However, the mechanism(s) underlying inflammaging remain elusive. Understanding the pathways that are responsible for inflammaging is of paramount importance in targeting interventions that can attenuate the age-associated increase in inflammation and improve healthspan.

Necroptosis and cellular senescence are cell fates that have been proposed to be involved in inflammaging [4]. Senescent cells release a highly heterogeneous secretome, collectively called senescence-associated secretory phenotype (SASP), which contains a wide array of proinflammatory factors, such as cytokines, chemokines, ROS, and extracellular matrix remodeling factors [6, 7]. SASP-factors can alter the behavior of neighboring cells [8], and their chronic presence can contribute to age-related diseases [9]. Although it is unclear whether the increase in cellular senescence with age is due to the generation of more senescent cells or the clearance of senescent cells is reduced with age [9], when the burden of senescent cells surpasses the rate of elimination, senescent cells accumulate in most tissues with age and contribute to prolonged inflammation [7]. Cellular senescence was originally characterized by the cessation of cell division when it was first discovered in vitro in the 1960s [10]. However, it is now known that the presence of senescent cells is observed in a variety of cells in vivo [11–14] and is associated with functional decline in various tissues [15]; therefore, it is recognized as one of the primary hallmarks of aging [2]. In contrast, necroptosis is a programmed cell death pathway that was officially defined by the Nomenclature Committee on Cell Death (NCCD) in 2018 [16]. Upon the initiation by necroptotic stimuli, such as TNFα, receptor-interacting serine/threonine-protein kinase (RIPK) 1 is activated, which results in the sequential phosphorylation of RIPK3 and mixed lineage kinase domain-like protein (MLKL), leading to MLKL oligomerization. The translocation of MLKL-oligomers to the plasma membrane and lysis of the cell results in the release of intracellular molecules, collectively called damage-associated molecular patterns (DAMPs), which are detected by pattern recognition receptors (PRRs) mainly expressed on innate immune cells to induce inflammation [1].

Our group was the first to show that necroptosis increased with age in various tissues of mice, e.g., adipose tissue [17], liver [18], and brain [19]. The age-related increase in necroptosis was associated with an increase in markers of inflammation in these tissues. Importantly, we showed that pharmacological suppression of necroptosis in the liver of old mice [18] and an age-accelerated mouse model [20] led to a reduction in inflammation, suggesting that necroptosis was a key driver in inflammation. To our surprise, inhibiting necroptosis also reduced cellular senescence markers [20, 21]. These data suggest that the age-related increase in necroptosis plays a role in the accumulation of senescent cells in older animals. To directly test the role of necroptosis in cellular senescence, we studied the impact of inducing necroptosis by the overexpression of MLKL in liver tissue of young/adult mice, which allowed us to explore the impact of MLKL overexpression induced necroptosis in the absence of confounding factors that accompany age, such as necroptosis, cellular senescence, oxidative stress, and inflammation. MLKL was overexpressed in hepatocytes of 2-month-old mice, and the effect of necroptosis on cellular senescence and inflammation was determined at 6 months of age. We found that the increase in necroptosis led to an increase in markers of cellular senescence, SASP-factors, inflammation, and fibrosis in the livers of 6-month-old mice.

## Methods

### Animals

The mice were generated and maintained in the animal facility at the Oklahoma City Veterans Affairs Health Care System Animal Facility. The *Mlkl* knock-in (KI) mice were generated on a C57BL/6J background as described previously [22]. Briefly, the transgene contained the cDNA to *Mlkl*, which was tagged with a Flag-tag (3x) sequence on the 3′-end of the cDNA and a stop cassette (3x) flanked by loxP sites inserted at the 5′ end of the transgene. The transgene was inserted into the intron region of the mouse ROSA26 locus between exon 1 and exon 2. Hemizygous *Mlkl*-KI female mice were crossed with male transgenic mice homozygous for the albumin-CreER^T2^ that were obtained from Dr. Youngmin Lee (Vanderbilt University Medical Center, Nashville, Tennessee). Approximately 50% of pups produced by this cross were Alb-CreER^T2^/*Mlkl*-KI mice as expected based on Mendelian inheritance. At 2 months of age, the Alb-CreER^T2^/*Mlkl*-KI mice were given 60 mg/kg body weight of tamoxifen (TMX from Sigma, St. Louis, MO) dissolved in corn oil (12 mg/mL) or corn oil by intraperitoneal injection for 5 consecutive days. Corn oil injected control mice were housed in separate cages to avoid carryover of TMX. TMX injection induces *Mlkl* expression specifically in hepatocytes in albumin-CreER^T2^/*Mlkl*-KI mice that was driven by the ROSA26 promoter (hereafter referred to as h*Mlkl*-KI mice). Figures S1A and B show there was no difference at 6 months of age in either the body weight or liver weight of the h*Mlkl*-KI mice compared to controls treated with corn oil (hereafter referred to as control mice). The mice were group housed (4 to 5 mice/cage) in ventilated cages at 20 ± 2 °C, on a12-h/12-h dark/light cycle, and fed a laboratory rodent chow (5053 Pico Lab, Purina Mills, Richmond, Indianapolis) ad libitum. Mice were euthanized 4 months after receiving the TMX or corn oil. Blood was collected in EDTA coated tubes and left undisturbed on ice for 15–30 min. Plasma was obtained by centrifuging the blood at 2000xg for 20 min at 4 °C, collecting the supernatant, and storing at −80 °C. Liver tissue was collected, frozen in liquid nitrogen, and stored at −80 °C until analyzed. All procedures were approved by the Institutional Animal Care and Use Committee at the Oklahoma City Veterans Affairs Health Care System Animal Facility.

### RNA isolation, quantification of mRNA transcripts, and transcriptomic analysis

Total RNA was extracted from liver using the RNeasy kit (Qiagen, Valencia, California) from 20 mg of frozen liver tissue as described previously [23]. RNA was used for RT-qPCR quantification of individual transcripts or quantification of the transcriptome using RNAseq. Reverse transcription PCR was performed using a high-capacity cDNA reverse transcription kit (Thermofisher Scientific, Waltham, Massachusetts) and RT-qPCR was performed with ABI Prism using Power SYBR Green PCR Master Mix (Thermofisher Scientific, Waltham, Massachusetts). The primers used for RT-qPCR analysis are given in Supplementary Table S1. The relative mRNA levels were determined by a series of calculations. First, the delta CT(∆CT) of the target gene was calculated by subtracting the CT value of the reference gene (β-microglobulin). Next, the delta delta CT (∆∆CT) was obtained by subtracting the ∆CT value of the target sample from the average of ∆CT values of the control samples. Finally, to calculate the fold changes in mRNA levels, we used the formula involving the exponentiation of 2 to the power of negative ∆∆CT (2^−ΔΔCt^). The fold change is determined by comparing the average ∆CT of the experimental group to the average of ∆CT of the control group.

RNAseq analysis was also performed on RNA isolated from liver using NEBNext Ultra II Directional RNA Library Prep Kit from Illumina (New England Biolabs; Massachusetts) as previously described (New England Biolabs: ref. 18). Paired-end 150 bp read sequencing was performed, in six biological replicates per group, on an Illumina NextSeq 500 sequencing platform. Raw reads, in a FASTQC format, were then imported into strand NGS (Next Generation Sequencing Analysis Software, version 4.0; Strand Life Sciences) for quality control, alignment to a reference genome, and statistical analysis. Sequences with a Q-score of < 30 were discarded, and the adaptor sequences were removed. Reads were then aligned the mouse reference genome (mm10), with both the genome build and gene annotations obtained from the UCSC Genome Browser (University of California, Santa Cruz). Data were then normalized and quantified using the DESeq2 algorithm inbuilt into the strand NGS software package. Read counts were log-transformed into “normalized signal values” and is used for further filtering, visualization, and statistical analysis [24, 25]. Transcripts with normalized signal values within a lower cutoff of 20.0 and upper cutoff of 100.0 (filtered by percentile) in ≥ 100% of samples in any 1 out of the 2 conditions were considered expressed at a level sufficient for quantification and transcripts outside of this range were excluded as previously described [24–27]. This filtering retained 24,989 genes, which were further used for differential expression analysis using a Z-test [28], with Benjamini–Hochberg correction applied to identify statistically significant genes (adjusted *p* < 0.05) showing a fold change ≥ 1.3. *p*-values appear as 0 in the Z-test results as shown in Supplementary Table S2, because of the numerical approximation used by strand NGS, where extremely low *p*-values are rounded down to 0 when they are below the precision limit.

Principal component analysis was performed and visualized using strand NGS (version 4) software. The RNAseq data was uploaded in MetaboAnalyst and data scaling was adjusted by Pareto scaling and genes were clustered, but samples were not, to generate heatmaps [29]. Pathways activated/inhibited were generated through the use of QIAGEN IPA (QIAGEN Inc., https://digitalinsights.qiagen.com/IPA) core analysis [30] using the significant differentially expressed genes. Cellular senescence gene set enrichment analysis (GSEA) was performed using GSEA 4.4.0 [31]. The gene sets were retrieved from the GSEA/the Molecular Signatures Database (MSigDB) [31, 32], and the sources of the gene sets include GO [33–35] Replicative senescence (GO:0090399), Reactome [36] [Cellular Senescence (R-MMU-2559583), Oncogene induced senescence (R-MMU-2559585), Oxidative stress induced senescence (R-MMU-2559580), DNA-Damage telomere stress induced senescence (R-MMU-2559586)], Kamminga et al*.* 2006 [37] (Kamminga senescence), and Saul et al*.* 2022 [38] (Saul Sen Mayo).

### Western blot

Western blots were performed as described previously [22]. Tissue (20 mg) was homogenized in HEPES lysis buffer (ThermoFisher Scientific, Waltham, Massachusetts) containing 2 mM phenylmethylsulfonyl fluoride and protease inhibitor cocktail (GoldBio, St Louis, Missouri). The protein concentration was determined using the Bio Rad BCA Protein Assay (Hercules, California). Western blotting was performed using 15–40 µg protein on an SDS-PAGE gel, and the protein was transferred onto a PVDF membrane. Images were taken using a Chemidoc imager (Bio-Rad, Hercules, California) and quantified using ImageJ software (U.S. National Institutes of Health, Bethesda, MD). The following primary antibodies were used: FLAG tag from Abcam (St.Louis, Missouri), RIPK3 from Novus biologicals (Centennial, Colorado), and MLKL, β-tubulin, and GAPDH from Sigma-Aldrich (St. Louis, Missouri). HRP-linked anti-rabbit IgG, HRP-linked anti-mouse IgG, and HRP-linked anti-rat IgG from Cell Signaling Technology (Danvers, Michigan) were used as secondary antibodies. To quantify the western blots, the intensity of the protein of interest was divided by the corresponding control band intensity (i.e., GAPDH or β-tubulin). Subsequently, the intensity of the band on each sample was then divided by the average intensity of the control group, thereby expressing the data as a fold change in each protein of interest.

The level of MLKL-oligomers was measured using western blots under non-reducing conditions as we have previously described [22]. Briefly, liver tissue was homogenized in HEPES buffer (pH 7.4), and protein samples were prepared using 2 × Laemmli buffer without any reducing agents to maintain the proteins under non-reducing conditions. Protein (40 μg) was applied to the gels, which were run under non-reducing conditions without SDS in the running buffer on 7.5% poly-acrylamide gels. MLKL-oligomers were measured using the MLKL antibody for oligomers larger than 200 kDa.

### Histological analysis of liver

Formalin-fixed liver tissue was embedded in paraffin and sectioned at 4 µm thickness using a microtome. Serial sections were placed on separate slides to allow for multiple types of staining on adjacent tissue sections. This approach allowed the direct comparison of histological features across different staining procedures, and the number of targets was measured in a field.

#### Immunohistochemical staining

The paraffin-embedded 4 µm liver sections were incubated with primary antibodies against Ki-67 (Abcam, Cambridge, Massachusetts) or αSMA (Thermoscientific, Waltham, Massachusetts) overnight at 4 °C. The diaminobenzidine-based colorimetric method was used for the detection of target proteins in the sections. Nuclei were counter stained with Mayer’s Hematoxylin (Sigma-Aldrich, St Louis, Missouri). Images were taken using a Zeiss Axioscanner (Zeiss, Dublin, California) for whole slide imaging, and three random fields per sample were used for analysis. TUNEL staining was performed with the paraffin-embedded 4 µm liver sections, using the DeadEnd™ Colorimetric TUNEL System (Promega, Madison, Wisconsin) according to the manufacturer’s instructions. Images of ×10 magnification (mononuclear cell cluster) and ×20 magnification (Ki-67, TUNEL, and αSMA) were used to quantify the targets using Image J software.

#### Picrosirius red (PSR) staining

PSR staining was conducted using formalin-fixed 4 µm sections that were deparaffinized and stained with PSR for 1 h. Excess PSR staining was removed by rinsing in acidified water, and sections were dehydrated with ethanol and cleared with xylene. The images were taken using a Zeiss Axioscanner (Zeiss, Dublin, California) for whole slide imaging, and three random fields per sample were used for analysis. Quantification was performed for 5–6 samples in each group using Image J software, expressed as the average percentage (%) of area. Using PSR-stained slides at ×20 magnification, the severity of hepatic fibrosis was quantified based on the Brunt scoring system [23]. This assessment was conducted as a double-blinded study with a total of 5–6 slides per group, and the slides were scored based on the presence of pathologic collagen using the following scoring system described in Figure S5. Thick collagen type I fibers and thin collagen type III fibers were detected and measured in the collagen deposits in liver from the PSR-stained slides using polarized microscopy. The images were taken with a Nikon TI Eclipse microscope (Nikon, Melville, New York), and three random fields per sample were used for analysis. Quantification was performed on 5–6 samples in each group using Image J software, normalized to PSR staining images, and expressed as the average percentage (%) of the stained area.

#### Masson’s trichrome (MT) staining

Staining was conducted using 4 µm sections of formalin-fixed liver tissue embedded in paraffin, which were deparaffinized and stained with MT for 1 h. Excess trichrome was removed by rinsing in acidified water, and sections were dehydrated with ethanol and cleared with xylene. The images were taken using a Zeiss Axioscanner (Zeiss, Dublin, California) for whole slide imaging, and three random fields per sample were used for analysis. Quantification was performed on ×20 magnification images of 5–6 samples in each group using Image J software, and the results are expressed as the average percentage (%) of area showing MT staining.

#### Histopathology score for inflammation

Hematoxylin and eosin (H&E) staining was performed on the tissue samples, and H&E-stained sections were digitally scanned using a Nikon Ti Eclipse microscope (Nikon, Melville, New York). Inflammatory foci were identified as a group of 10–12 mononuclear cells together and counted manually. The number of cell clusters was quantified by counting the number of inflammatory foci/clusters per field using ×10 magnification. This assessment was performed double blinded, and 5–6 slides per group were scored for inflammatory foci/clusters. Five different fields were counted, and the average was subsequently scored using the following categories: 0 (none), 1 (< 2 foci), 2 (2–4 foci), 3 (> 4 foci).

### Assays for plasma alanine transaminase (ALT) and high mobility group box 1 (HMGB1)

The activity of ALT in plasma was measured using the alanine transaminase colorimetric activity assay kit from Cayman Chemical Company (Ann Arbor, Michigan) following the manufacturer’s instructions and expressed as U/L. Plasma levels of HMGB1 were measured using the mouse HMGB1 ELISA Kit (R&D Systems, Minneapolis, Minnesota) as per the manufacturer’s instructions and expressed as ng/mL.

### Statistical analysis

To determine the appropriate statistical method to compare two groups, we first performed the Shapiro–Wilk normality test. If a group failed to pass the normality test, the comparison was made using the Mann–Whitney *U* test. If both groups passed the normality test and the equality of variances between the groups was not violated, the groups were subjected to Welch’s *t*-test. Otherwise, the groups were compared using the two-tailed Student unpaired *t*-test. The alpha value was set at 0.05, rendering *p*-values below this value to be considered statistically significantly different.

## Results

### MLKL overexpression in the liver leads to necroptosis and liver damage

**Fig. 1:**
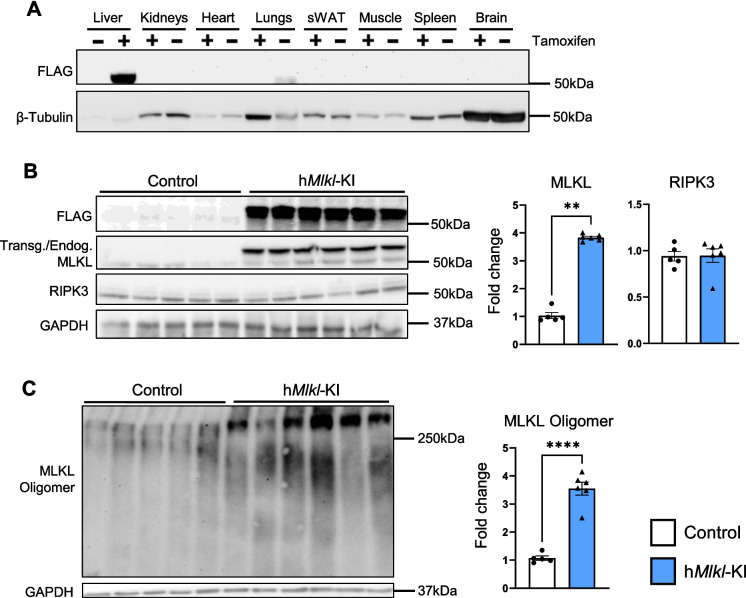
MLKL is overexpressed in the livers of h*Mlkl*-KI mice upon TMX treatment. At 2 months of age, Alb-CreER^T2^/*Mlkl*-KI mice were treated with corn oil or TMX, and the livers were collected from the mice at 6 months of age. **A** Western blot showing the expression of FLAG-tagged MLKL in various tissues of the Alb-CreER^T2^/*Mlkl*-KI mice treated with corn oil (-) or TMX (+). **B** Western blot showing the levels of FLAG-tag, MLKL from the exogenous transgene (upper band) or endogenous gene (lower band), and RIPK3 in the livers of control and h*Mlkl*-KI mice. **C** Native PAGE MLKL western blot showing the level of MLKL oligomers in the livers of control and h*Mlkl*-KI mice. The graphs quantifying the levels of MLKL and RIPK3 and MLKL-oligomers in the Alb-CreER^T2^/*Mlkl*-KI mice treated with corn oil (white bars) or TMX (blue bars) are shown for each animal as well as the mean ± SEM for 5 to 6 mice per group. Statistical significance was assessed by Mann–Whitney *U* test (for MLKL and RIPK3) and Welch’s *t*-test (for MLKL oligomers). **p < 0.01, ****p < 0.0001

To induce necroptosis specifically in the liver, we crossed *Mlkl*-KI mice to mice that express tamoxifen-inducible Cre recombinase driven by the albumin promoter. Figure 1 shows that Alb-CreER^T2^/*Mlkl*-KI mice express a transgene containing the FLAG-tag under the control of the ROSA26 promoter when they are treated at 2 months of age with tamoxifen (TMX), designated as h*Mlkl*-KI mice. The expression of the *Mlkl*-transgene (FLAG-tagged) is present only in the livers of TMX-treated mice and is not expressed in the control mice treated with corn oil, as would be expected, because the Alb-CreER^T2^-transgene is expressed by hepatocytes [39]. We assume that all hepatocytes would express the MLKL transgene because the albumin-CreER^T2^ and the *Mlkl*-KI transgenes would be expressed in all hepatocytes. Figure 1B shows that at 6 months of age, the levels of MLKL are increased ~ 4-fold in the h*Mlkl*-KI mice compared to control mice. However, there was no significant difference in the RIPK3 levels in the liver (Fig. 1B), body weight (Figure S1A), and liver weight (Figure S1B) between the h*Mlkl*-KI and control mice.

**Fig. 2:**
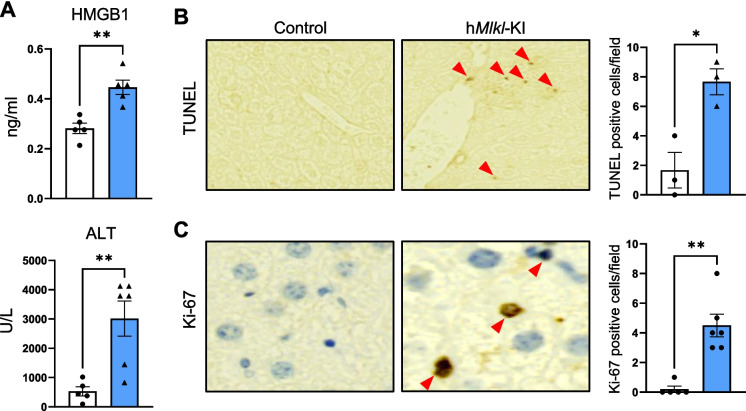
Hepatic MLKL overexpression induces necroptosis, cell death, and damage. Markers of necroptosis and liver damage were measured in the livers and plasma collected from 6-month-old control and h*Mlkl*-KI mice. **A** HMGB1 levels and ALT activity measured in the plasma. **B** Representative TUNEL staining images (×20 magnification) as well as the number of TUNEL-positive cells in the liver and the graph showing the number of TUNEL-positive cells/field. **C** Representative Ki-67 staining images (×40 magnification) showing the Ki-67 positive cells (where Ki-67 is colocalized with the nucleus as shown by the arrows) in the liver and the graph showing the number of Ki-67 positive cells/field. An average of 19% of the cells from the livers of the h*Mlkl*-KI mice stain positive for Ki-67 compared to an average of 6% of the cells from control livers. The graphs show data from individual control (white bars) and h*Mlkl*-KI (blue bars) mice as well as the mean ± SEM for 5 to 6 mice per group, except for the TUNEL assay, which was conducted with 3 animals in each group. Statistical significance was assessed by two-tailed Student’s unpaired *t*-tests (for HMGB1 and TUNEL positive cells) and Mann–Whitney *U* test (for ALT activity and Ki-67 positive cells). *p < 0.05, **p < 0.01

To investigate whether MLKL overexpression leads to necroptosis in the liver, we first measured the presence of MLKL-oligomers in the liver, which is essential for plasma membrane permeabilization and necroptosis [16]. Figure 1C shows a 3- to 4-fold increase in MLKL-oligomers in the livers of 6-month-old h*Mlkl*-KI mice compared to control mice. We further validated an increase in necroptosis in the h*Mlkl*-KI mice by measuring plasma levels of HMGB1 and ALT activity. Upon plasma permeabilization, necroptotic cells release DAMPs, such as HMGB1[1]. We found a ~ 60% increase in HMGB1 levels (Fig. 2A) in the plasma from h*Mlkl*-KI mice. The h*Mlkl*-KI mice also showed a ~ 8-fold increase in ALT activity in the plasma, which is a measure of liver damage and cell death [40]. To further characterize the liver damage induced by overexpressing MLKL, we performed TUNEL and Ki-67 staining. TUNEL staining detects necroptotic cells [41] and was increased ~ 4.5-fold in the livers of the h*Mlkl*-KI mice (Fig. 2B). Ki-67 staining detects proliferating cells [42], which would be expected in response to the death of hepatocytes. The h*Mlkl*-KI mice showed a ~ 22-fold increase in Ki-67 positive cells (Fig. 2C) compared to control mice. Thus, the data in Figs. 1 and 2 show that overexpressing MLKL in the livers of young/adult 6-month-old mice induces necroptosis, which is normally negligible in the liver at 6 months of age.

### MLKL overexpression leads to increased inflammation in the liver

**Fig. 3:**
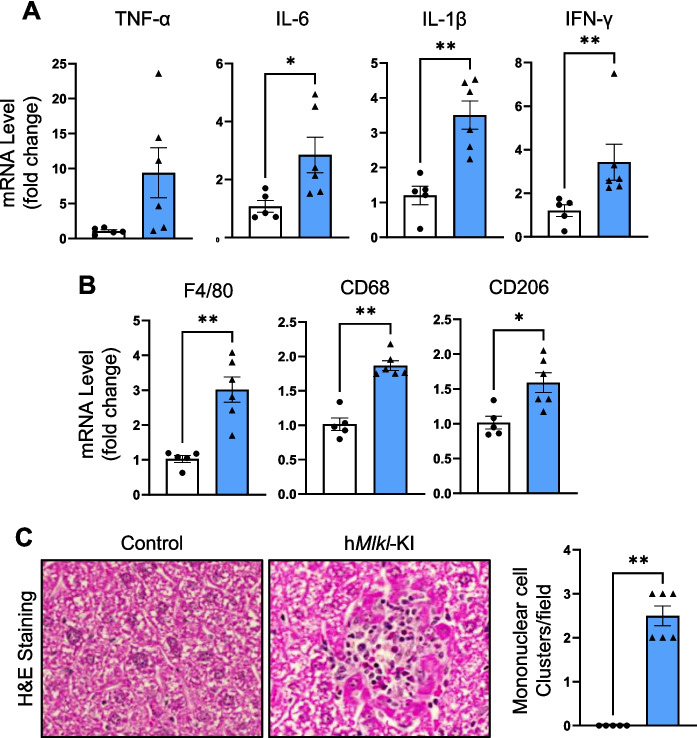
Hepatic MLKL overexpression leads to inflammation in the liver. Various markers of inflammation were measured in the livers of 6-month-old control and h*Mlkl*-KI mice. **A** Transcript levels of proinflammatory cytokines. **B** Transcript levels of macrophage markers. **C** Representative H&E images (×40 magnification) of the liver showing the presence of a mononuclear cell cluster in TMX-treated mice and the quantification of mononuclear cell clusters/field. The graphs show data from individual control (white bars) and h*Mlkl*-KI (blue bars) mice as well as the mean ± SEM for 5 to 6 mice per group. Statistical significance was assessed by Welch’s *t*-test (for TNF-α, IL-6), two-tailed Student’s unpaired *t*-test (for IL-1β, CD206), and Mann–Whitney *U* test (for IFN-γ, F4/80, CD68, mononuclear cell clusters). *p < 0.05, **p < 0.01

Because necroptosis is a cell fate that is involved in chronic inflammation, we next assessed the effect of MLKL overexpression on inflammation in 6-month-old mice by measuring the expression of proinflammatory cytokines and macrophage levels, which are recruited to the site of inflammation in response to increased chemoattractants and cytokines [43]. Figure 3A shows that transcript levels of IL-6, IL-1β, and IFN-γ were significantly elevated in the livers of h*Mlkl*-KI mice compared to control mice. TNF-α levels were increased over 8-fold; however, this increase was not statistically significant because of the variability in the h*Mlkl*-KI mice. Figure 3B shows that the transcript levels of markers for pan-macrophages (F4/80), M1-macrophages (CD68), and M2-macrophages (CD206) were significantly higher in the livers of h*Mlkl*-KI mice. In addition, we performed H&E staining to measure the number of mononuclear cell clusters (Fig. 3C), which is indicative of inflammation [23], particularly chronic inflammation [44]. We observed 2 to 3 mononuclear cell clusters per field in the livers of h*Mlkl*-KI mice, while no clusters were observed in the livers of the control mice. These data show that necroptosis induced by MLKL overexpression leads to inflammation in the liver at an age when age-related increases in inflammation are minimal.

### MLKL overexpression induces cellular senescence in the liver

**Fig. 4:**
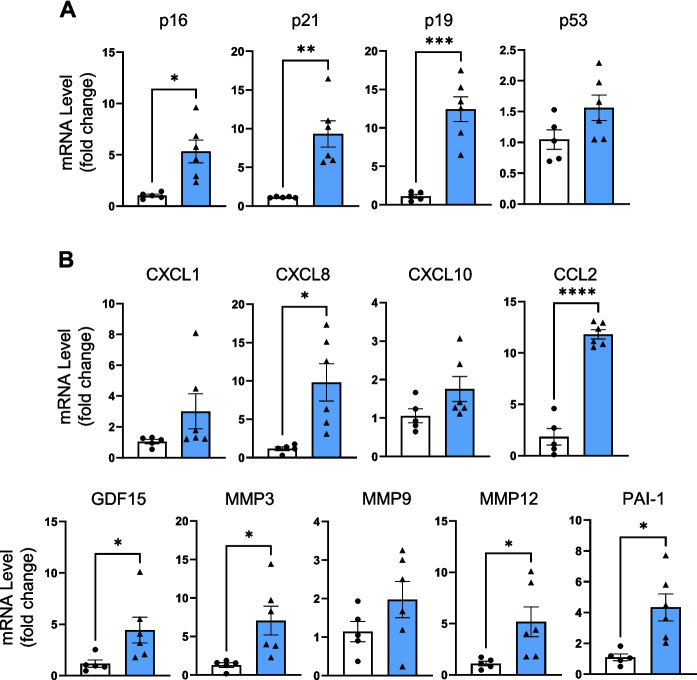
Hepatic MLKL overexpression increases cellular senescence. Transcript levels of cell cycle arrest proteins (**A**) and SASP factors (**B**) in the livers of control (white bars) and h*Mlkl*-KI (blue bars). The data are shown for individual animals as well as the mean ± SEM for 5 to 6 mice per group. Statistical significance was assessed by Welch’s *t*-test (for p16, p21, p19, CXCL8, GDF15, MMP3, MMP12, PAI-1), two-tailed Student’s unpaired *t*-tests (for p53, CXCL10, CCL2, MMP9), and Mann–Whitney *U* test (for CXCL1). *p < 0.05, **p < 0.01, ***p < 0.001, ****p < 0.0001

Our previous studies suggested that increased necroptosis was associated with increased cellular senescence [18, 20]; therefore, we determined if inducing necroptosis in 6-month-old mice had an impact on cellular senescence. We assessed several markers of cellular senescence in the livers of h*Mlkl*-KI and control mice because there is not a single unequivocal marker of cellular senescence. However, cell cycle arrest factors, especially p16^INK4A^ and p21^Cip1/Waf1^, are widely touted as the most salient markers. We observed that the transcript levels of p16 and p21 were significantly increased by 5-fold and 9-fold (Fig. 4A), respectively, in the h*Mlkl-*KI mice. We also observed that p21 protein levels were significantly increased in the h*Mlkl*-KI mice (Figure S1C). In addition, transcript levels of other proteins involved in the cell cycle arrest pathways, such as p53 and p19, were also increased; however, this increase was only significant for p19 (Fig. 4A).

Because SASP factors are a hallmark of cellular senescence and have been proposed to contribute to chronic inflammation, we measured the transcript levels of nine SASP-factors commonly upregulated in senescent cells [45] as shown in Fig. 4B. While not all the SASP factors we measured were significantly increased, all of them exhibited upregulation in the livers of the h*Mlkl*-KI mice (Fig. 4B). For example, transcript levels of chemokines, such as CXCL and CCL family members, were increased by 1.5 to 8.5-fold, transcript levels of GDF-15, a growth differentiation factor, were increased by 4-fold, and transcript levels of extracellular matrix proteases/regulators, such as MMP3, MMP9, MMP12, and PAI-1, were increased from 1.7- to 5-fold. These data clearly demonstrate that inducing necroptosis in the livers of young/adult mice also induces cellular senescence at an age when cellular senescence is minimal/undetectable.

### MLKL overexpression alters hepatic transcriptome

**Fig. 5:**
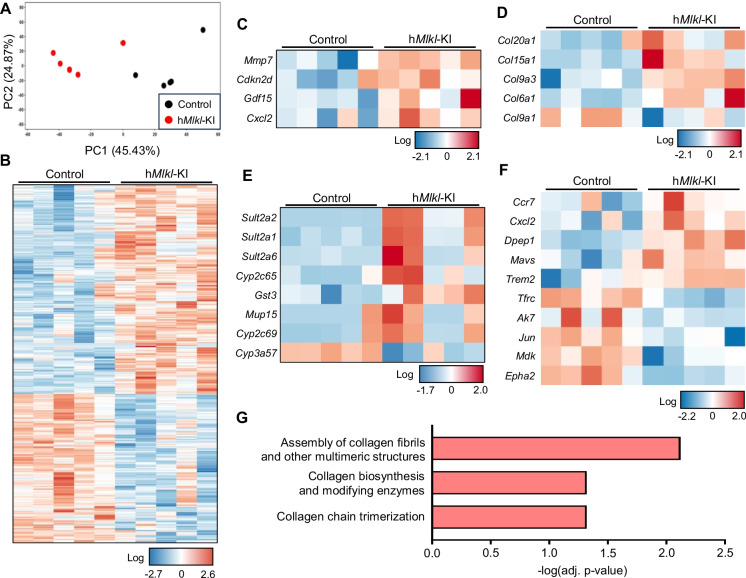
MLKL overexpression alters the transcriptome of the liver. RNAseq data were obtained from the livers of five h*Mlkl*-KI mice and five control mice at 6 months of age. **A** PCA plot showing that the control and h*Mlkl*-KI mice are separately clustered. **B** The heatmap plot showing the relative expression of the 420 DEGs significantly (cutoffs of FC ≥ 1.3 and adj. *p*-value  < 0.05) altered in the livers of each of the h*Mlkl*-KI and control mice. The relative expression levels of DEGs involved in cellular senescence (**C**), collagenase (**D**), drug metabolism (**E**), and inflammation (**F**) are shown in heatmaps. **G** Pathways significantly (adj. *p*-value < 0.05) upregulated in h*Mlkl*-KI using IPA

To further explore the biological impact of necroptosis induced by hepatic MLKL overexpression, we compared the transcriptome of liver from h*Mlkl*-KI and control mice. Principal component analysis (PCA) of the transcriptome patterns showed a clear clustering of samples with a separation between control and h*Mlkl*-KI mice (Fig. 5A), indicating that the transcriptome of the liver was altered by overexpressing MLKL. Using FC ≥ 1.3 and Benjamini-Hochberg (BH) adjusted (adj.) *p*-value < 0.05 cutoffs, we found a total of 420 differentially expressed genes (DEGs) in the livers of the h*Mlk*l-KI mice, 175 downregulated DEGs and 245 upregulated DEGs (listed in Supplementary Table S2). The relative expression of the 420 DEGs is shown in Fig. 5B for each mouse. As expected, we found the expression of several genes involved in cellular senescence was increased 30 to 170% (Fig. 5C). For example, the cell cycle arrest gene, *Cdkn2d* (p19), as well as several SASP-factors, such as *Mmp7*, *Cxcl2*, and *Gdf15*, which agree with the RT-qPCR data in Fig. 4. In addition, the levels of several collagenase transcripts were increased 30 to 70% (Fig. 5D), which encode proteins that could potentially contribute to a buildup of collagen fibers in the liver and to fibrosis. As shown in Fig. 5E, we also found an increase in transcript levels of several genes involved in drug metabolism in phase I (*Cyp*’s) and phase II (*Sult*’s and *Gst3*) and the elimination of the metabolites in Phase III (*Mup15*). Particularly striking was the dramatic upregulation (~ 200- and ~ 600-fold) of the sulfotransferases, *Sult2a1* and *Sult2a2*, respectively, which are involved in the sulfation of hydrophobic molecules making them more water-soluble and easier to eliminate.

Because data in Fig. 3 showed an increase in inflammation in response to increased necroptosis, we determined if any of the DEGs were associated with inflammation. The data in Fig. 5F show that 10 of the 420 DEGs were involved in inflammation. We found two of the genes (*Ak7* and *Cd276*), which suppress inflammation [46, 47] were reduced ~ 40%, and five of the genes, which are associated with increased inflammation, were increased 30 to 80% in the h*Mlkl*-KI mice. For example, MAVS induces activation of NF-kB and promotes proinflammatory cytokine production [48, 49], DPEP1 acts as a major adhesion receptor for neutrophils [50] and is involved in the recruitment of inflammatory monocytes [51], and TREM2 is expressed on immune cells, such as macrophages and neutrophils, which infiltrate the liver in response to injury [52]. CXCL2 is a chemokine, also known as macrophage inflammatory protein 2, that promotes the recruitment of neutrophils [53], and CCR7 is a chemokine receptor that is upregulated in leukocytes, such as dendritic cells, and mediates adhesion and migration to secondary lymphoid organs [54, 55]. These data support our previous data (Fig. 3) indicating that the overexpression of MLKL induced inflammation in the liver. On the other hand, three of the downregulated DEGs would be expected to limit inflammation, e.g., TFRC promotes infiltration and activation of macrophages [56], MDK is involved in the NF-κB pathway activation [57], EphA2 promotes monocyte adhesion [58], and Jun plays a role in macrophage activation [59]. However, MDK, Jun, and EphA2 are also involved in cell proliferation and cell cycle arrest. For example, suppression of *Mdk* suppresses p-CDK1 and induces G2/M cell cycle arrest [60] and knockout of *Jun* and inactivation of Jun/JNK induces p53-independent cell cycle arrest and has been proposed as a potential antitumor intervention [61]. The expression of *Epha2* is elevated in cancer cells and genetic inhibition of *Epha2* induces cell cycle arrest [62]. Thus, we speculate that the decrease in these genes plays a role in the induction of cell cycle arrest and therefore, contributes to cellular senescence, supporting our data in Fig. 4 that cellular senescence is induced by necroptosis.

We first performed Ingenuity Pathway Analysis (IPA) on the 420 DEGs to identify potential biological pathways that could be altered by overexpressing MLKL in the liver. As shown in Figure S2, we found 20 pathways that reached a threshold (*p* < 0.05), and three pathways shown in Fig. 5G are significantly (adj. *p* < 0.05) upregulated in h*Mlkl*-KI mice after post-hoc analysis. These pathways include (1) assembly of collagen fibrils and other multimeric structures, (2) collagen biosynthesis and modifying enzymes, and (3) collagen chain trimerization. In addition, the next two ranked pathways that did not reach the BH-corrected significance were collagen degradation and extracellular matrix organization. All five pathways are involved in collagen turnover and structure and could potentially contribute to fibrosis as well as the thirteenth pathway on the list, pulmonary fibrosis idiopathic signaling. We also performed Gene Set Enrichment Analysis (GSEA) for KEGG (Kyoto Encyclopedia of Genes and Genomes) and GO (Gene Ontology) pathways on the RNAseq; however, no pathways were found to differ significantly (*p* < 0.05). We also interrogated the RNAseq data using seven cellular senescence gene sets publicly available in the GSEA/the Molecular Signatures Database (MSigDB) [31, 32]. As shown in Figure S3, a trend for an increase in cellular senescence (NES 0.9480 to 1.1632) was observed for the h*Mlkl*-KI mice in all seven gene sets; however, these increases were not significant at the *p* < 0.05 level. Interestingly, one of the IPA twenty pathways listed in Figure S2 that was altered by necroptosis in the liver was the wound healing signaling pathway, which was upregulated and is a pathway that requires the transient presence of senescent cells to restore tissue architecture.

### MLKL overexpression leads to fibrosis in the liver

**Fig. 6:**
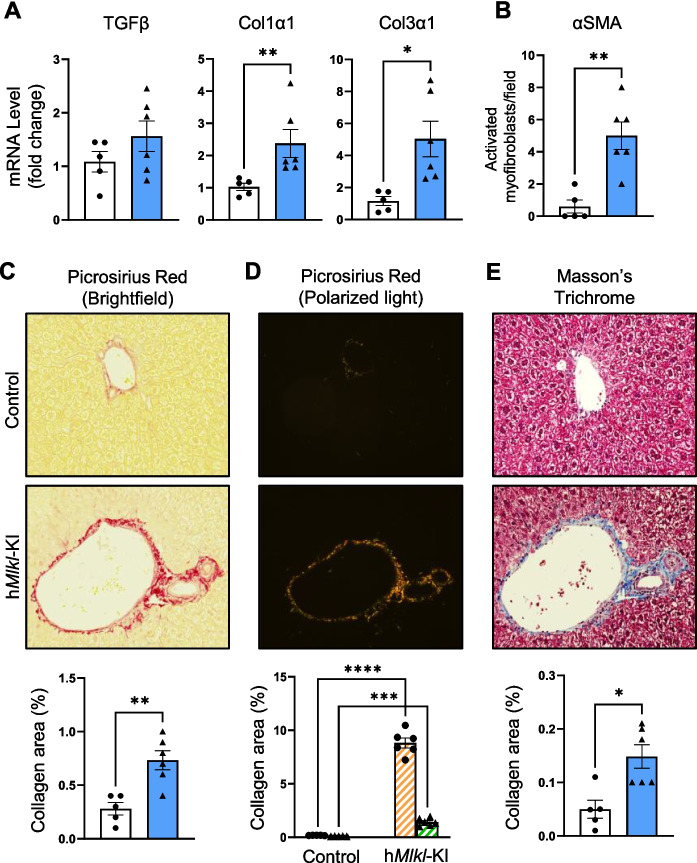
MLKL overexpression leads to fibrosis in the liver. Various markers of fibrosis were measured in the livers of control and h*Mlkl*-KI mice at 6 months of age. **A** Transcript levels of fibrosis markers (TGFβ, Col1α1, and Col3α1). **B** The number of activated myofibroblasts in the liver (representative images of αSMA staining are shown in Figure S4A). **C** Representative PSR staining images (×20 magnification) showing the deposition of collagen (red staining) in the portal triad and the quantification of the percentage area staining for collagen/field. **D** Representative images of the birefringence exhibited by collaged fibers of PSR-stained liver tissue (×20 magnification) under polarized light. The yellow-orange staining shows the presence of thick collagen type I fibers and green staining shows the presence of thin collagen type III fibers, which are shown in the graph as the percentage area for type I fibers (orange bars) or type III fibers (green bars) per field. **E** Representative images of MT staining (×20 magnification) showing the deposition of collagen (blue staining) in the portal triad and the quantification of the percentage area staining for collagen/field. The images in Figure S4B show representative images of PSR and MT staining of whole liver slices. The graphs show data from control (white bars) and h*Mlkl*-KI (blue bars) mice presented as the mean ± SEM for 5 to 6 mice per group with data shown for individual mice. Statistical significance was assessed by Welch’s *t*-test (Col3α1, and type I and type III collagen fibers), two-tailed Student’s unpaired *t*-tests (TGFβ, PSR-stained collagen area), and Mann–Whitney *U* test (αSMA, and Col1α1 and MT-stained collagen area). *p < 0.05, **p < 0.01

Because we observed an increase in the expression of several collagenase genes and pathways involved in collagen formation and because inflammation plays a major role in fibrosis [63], we wanted to determine if inducing necroptosis in 6-month-old mice led to liver fibrosis at an age when fibrosis was minimal. We first measured the expression levels of the most studied and robust markers of fibrosis [23, 64, 65]. Figure 6A shows that transcripts for TGF-β, Col1α1, and Col3α1 were all increased at 6 months of age in the livers of h*Mlkl*-KI mice; however, the increase for TGFβ was not statistically significant. We also measured the number of cells exhibiting colocalization of the nucleus with α-smooth muscle actin (αSMA), which is a key marker for myofibroblasts that transdifferentiate from activated hepatic stellate cells and produce extracellular matrix proteins, such as collagen [66]. As shown in Fig. 6B, the levels of myofibroblasts were increased 8-fold in the h*Mlkl*-KI mice compared to the control.

While the biomarkers indicated that fibrosis was increased in the livers of h*Mlkl*-KI mice, we established the presence of fibrosis in the liver using pathological techniques. Therefore, we histologically assessed fibrosis in the livers of h*Mlkl*-KI and control mice using either picrosirius red (PSR) or Masson’s trichrome (MT) staining for collagen deposition. Typically, collagen deposition in the liver begins around the portal triads, forming fibrous septa that can extend outward into the lobular parenchyma as the fibrosis progresses. Figure 6C shows PSR staining of the collagen deposition in the liver, which was localized primarily to the portal triad in the liver of h*Mlkl*-KI mice. This was rarely observed in the control mice (Figure S4B). When measuring the percentage of PSR staining area in the liver tissue, we found a  ~ 2.5-fold increase in PSR staining in the h*Mlkl*-KI mice (graph in Fig. 6C). Because fibrosis involves an increase in thick collagen type I, which leads to an increase in thin collagen type III fibers [67], we used polarized microscopy combined with PSR staining to determine if these types of fibers were present in the collagen deposits in the h*Mlkl*-KI mice. As shown in Fig. 6D, the thick collagen type I fibers and thin collagen type III fibers were dramatically increased in the collagen deposits of h*Mlkl*-KI mice. This is likely due to increased crosslinking of collagen in h*Mlkl*-KI mice compared to the control mice. We also used MT staining to detect and quantify collagen deposition in the livers of the 6-month-old mice. The images in Figs. 6E and S4B show increased collagen deposition, which is again localized primarily to the portal triad in the liver from the h*Mlkl*-KI mice. A 3-fold increase in the percentage of collagen area in the livers of h*Mlkl*-KI mice was observed with MT staining. We also assessed the severity of hepatic fibrosis in the PSR staining slides using the Brunt scoring system, which is based on the presence of pathologic collagen ranging from 0 (none) to 4 (cirrhosis). As shown in Figure S5, the Brunt score was higher for the h*Mlkl*-KI mice, ranging from 1 to 3, compared to 0 to 1 for control mice. These data demonstrate that MLKL overexpression leads to the initial stages of fibrosis in the liver at 6 months of age, before fibrosis would normally develop.

## Discussion

Over the past decade, most of the research in aging has focused on how senescent cells contribute to aging and the impact of eliminating senescent cells on aging. Less is known about the factors that occur during aging that are responsible for inducing cells to undergo senescence. The initial studies of Hayflick [10] showed that primary cells in culture underwent senescence after a finite number of cell doublings. Subsequently, the laboratories of Wright and Shay [68] showed that telomere erosion, which occurred as the cells proliferated, was responsible for cells undergoing senescence in culture. This discovery was key in showing that DNA damage arising from the loss of telomeres triggered stable cell cycle arrest, preventing the DNA-damaged cells from proliferating and possibly becoming cancerous. Subsequent studies showed that DNA damage could directly lead to cellular senescence in animals, e.g., treating animals with DNA-damaging agents such as doxorubicin [69–71] and radiation [72] and knocking down genes involved in DNA repair, such as BRCA1 and BLM [73]. In addition, other factors have been shown to trigger cellular senescence in tissues of animals, e.g., oxidative stress (e.g., *Sod1*-KO mice [74]), oncogene activation (e.g., TRAS mice [75, 76]) mitochondrial dysfunction (e.g., POLG^D257A^ mice [77]), and epigenetic alterations (e.g., ICE system mice [78]).

When studying the role of necroptosis in aging and inflammaging, we were surprised to find that when necroptosis was pharmacologically inhibited in the livers of old [18] or *Sod1*-KO [20] mice, cellular senescence was also reduced, suggesting that necroptosis might play a role in inducing cellular senescence as animals aged. However, it was also possible that necroptosis in these studies was indirectly increasing senescence through its actions on aging. In this study, we directly tested the role of necroptosis in cellular senescence, using a novel mouse model we generated that allowed us to overexpress MLKL in the hepatocytes of young/adult mice when factors like DNA damage, oxidative stress, and mitochondrial dysfunction are minimal. We showed that inducing MLKL expression (~ 4-fold) increased necroptosis in the liver as demonstrated by MLKL-oligomerization, increased levels of HMGB1 in plasma, and markers of liver cell death/damage. Although all hepatocytes will likely overexpress MLKL, we believe that only a subset of hepatocytes will be activated to undergo necroptosis in response to stress based on our previous study and the observation that liver weight did not change in the h*Mlkl*-KI mice (Figure S1B). Using the presence of proliferating cells (Fig. 2C) as an indirect measure of hepatocytes replacing the hepatocytes lysed and cleared, we speculate that ~ 15% of the hepatocytes in the h*Mlkl*-KI mice are undergoing necroptosis. The induction of necroptosis triggered a 5- to 9-fold increase in transcript levels of CDK inhibitors involved in cell cycle arrest, such as p16^INK4A^ and p21^Cip1/Waf1^, as well as different types of SASP-factors, including proinflammatory cytokines, chemokines, growth differentiation factors, and extracellular matrix proteases/regulators. In addition, transcriptomic analysis revealed that inducing necroptosis in the young/adult mice also altered several genes involved in cellular senescence as well as genes (*Mdk, Jun,* and *Epha2*) that negatively regulate cell cycle arrest [60–62], which could potentially lead to cellular senescence in the livers of h*Mlkl*-KI mice.

As expected, because necroptosis is a key pathway in inducing inflammation, we found that 6-month-old h*Mlkl*-KI mice also showed an increase in transcript levels of various markers of inflammation (Figs. 3 and 5F). These increases in necroptosis, cellular senescence, and markers of inflammation were associated with an increase in fibrosis, as measured by collagen deposition and fibrosis severity (Brunt score). Although the induction of fibrosis was associated with an increase in necroptosis and cellular senescence, it is unclear whether either one of the cell fates or both the cell fates were responsible for fibrosis because the activation of hepatic stellate cells, which causes the buildup of extracellular matrix components [79], can be triggered by both necroptotic liver damage and SASP factors, such as TGF-β [80].

While our data conclusively demonstrate that inducing necroptosis in vivo can trigger cellular senescence in the liver, the next question is how this occurs? We envision necroptosis inducing cellular senescence in two ways. First, through a cell-autonomous mechanism where the overexpression of MLKL induces the cells to undergo cellular senescence. Once MLKL is phosphorylated and oligomerizes, it has been shown that the positively charged residues of MLKL interact with negatively charged phosphatidylinositol phosphates (PIPs) of the plasma membrane, resulting in the lysis of the cell [81]. In our study, at least a subset of cells expressing transgenic MLKL remain after the 4 months, which implies that there are cells resistant to necroptotic cell death even when overexpressing MLKL. It is possible that these cells induce cellular senescence in a cell-autonomous manner. For example, MLKL-oligomers might bind and disrupt the integrity of mitochondrial membranes that could lead to mitochondrial dysfunction, which has been shown to induce cellular senescence [77]. In addition, MLKL oligomers can also bind and disrupt the autolysosomal membranes that can lead to inhibition of autophagic flux [82], which could lead to cellular senescence because autophagy has been shown to suppress cellular senescence [83]. However, this mechanism seems unlikely, because autophagy has also been reported to promote cellular senescence through TOR-autophagy spatial coupling compartment (TASCC), which stimulates the protein synthesis of SASP-factors [84]. Because necroptosis is a cell death pathway, it would seem more likely that necroptosis would lead to the elimination of senescent cells through a cell-autonomous mechanism rather than increasing them. This interpretation is consistent with our previous studies showing pharmacological inhibition of necroptosis in old [18] and *Sod1*-KO [20] mice decreased senescence rather than increasing cellular senescence.

A second possible mechanism, which we believe is more likely, is necroptosis induces cellular senescence through a cell-non-autonomous mechanism. This could occur in two ways from DAMPs released from necroptotic cells undergoing lysis. First, DAMPs released by necroptotic cells induce neighboring cells to become senescent. It is well established that the DAMP, HMGB1 released by necroptotic cells, is recognized by pattern recognition receptors (PRRs) [85] and its downstream signaling mediates the activation of immune response pathways. The NF-κB pathway is one of the immune response pathways triggered by activation of PRRs [86], which is also a central regulator of SASP-factors [87]. Importantly, overexpression of HMGB1 has been shown to promote cellular senescence in cultured cells [88]. In addition, when senescent cells were cultured with HMGB1-blocking antibody, the secretion of IL-6 was attenuated [88]. IL-6 is a SASP-factor that has been shown to induce a self- and cross-reinforcement of cellular senescence [89]. A second mechanism of how DAMPs released from necroptotic cells could impact cellular senescence is through the elimination of senescent cells. One well-characterized mechanism by which senescent cells are removed is macrophage-mediated clearance [90]. Interestingly, activated macrophages induced by DAMPs are reported to have reduced phagocytic activity. For example, HMGB1 released by necroptotic cells has been reported to impair the phagocytic activity of lung macrophages [91, 92]. Thus, the increase in cellular senescence we observed in the livers of the h*Mlkl*-KI mice could also arise from the reduced clearance of senescent cells because of reduced phagocytic activity of macrophages. On the other hand, the decrease in cellular senescence that we observed in the livers of old mice [18] or *Sod1*-KO mice [20] treated with Nec-1s could arise from an increased removal of senescent cells because of increased phagocytic activity of macrophages arising from the inhibition of necroptosis by Nec-1s.

In summary, our study clearly demonstrates that necroptosis can induce cellular senescence in vivo when other factors known to induce cellular senescence (e.g., DNA damage, oxidative stress, and mitochondrial dysfunction) are minimal. Our study also suggests that an interaction between the two cell fates could play an important role in inflammaging. For example, it is well documented that necroptotic cells trigger a major, immediate inflammatory storm when DAMPs released by the dying cells are recognized by PRRs expressed on innate immune cells. Necroptosis could also have a long-term impact on inflammation through cellular senescence. Senescent cells induced by necroptosis would continuously secrete SASP factors that could lead to inflammation until senescent cells are eventually cleared by the immune system, which would occur well after the necroptotic event. Thus, using a combination of drugs to inhibit both necroptosis and clear senescent cells has the potential of being effective in the long-term treatment of inflammaging. Several inhibitors of necroptosis are currently available that are FDA approved (e.g., necrostatin-1s (Nec-1s), ponatinib, pazopanib, dabrafenib, and sorafenib), and several senolytics are under development and testing in humans. For example, over 20 senolytic clinical trials are currently being conducted to target various age-related diseases, such as idiopathic pulmonary fibrosis, as well as Alzheimer’s and chronic kidney diseases [7]. Therefore, a combination of these therapeutic strategies that are translatable to humans could reduce chronic inflammation in the elderly and improve healthspan.

## Supplementary Information

Below is the link to the electronic supplementary material.

ESM 1 (PDF 586 KB)

ESM 2 (DOCX 29.3 KB)

ESM 3 (DOCX 18.1 KB)

ESM 4 (XLSX 88.2 KB)

## Data Availability

The data supporting the findings of the study are available in the manuscript and supplementary material of this article. Correspondence and requests for information should be addressed to A.R.

## Abbreviations

Ripk1: Receptor-interacting protein kinase 1
Ripk3: Receptor-interacting protein kinase 3
Mlkl: Mixed lineage kinase domain-like
DAMPs: Damage-associated molecular patterns
SASP: Senescence-associated secretory phenotype
ALT: Alanine aminotransferase
H&E: Hematoxylin and eosin
HMGB1: High mobility group box 1
ROS: Reactive oxygen species
PRRs: Pattern recognition receptors
PSR: Picrosirius red
MT: Masson’s trichrome
TMX: Tamoxifen
