# Supplementary material for: Necroptosis induced by MLKL overexpression in liver triggers cellular senescence and leads to chronic inflammation and fibrosis
Source: GeroScience. 2025 Nov 24;48(2):1917–35. doi: 10.1007/s11357-025-01994-y (PMC12972278; doi:10.1007/s11357-025-01994-y)
Supplement: Supplementary file 1 — (PDF 586 KB) [file 11357_2025_1994_MOESM1_ESM.pdf]

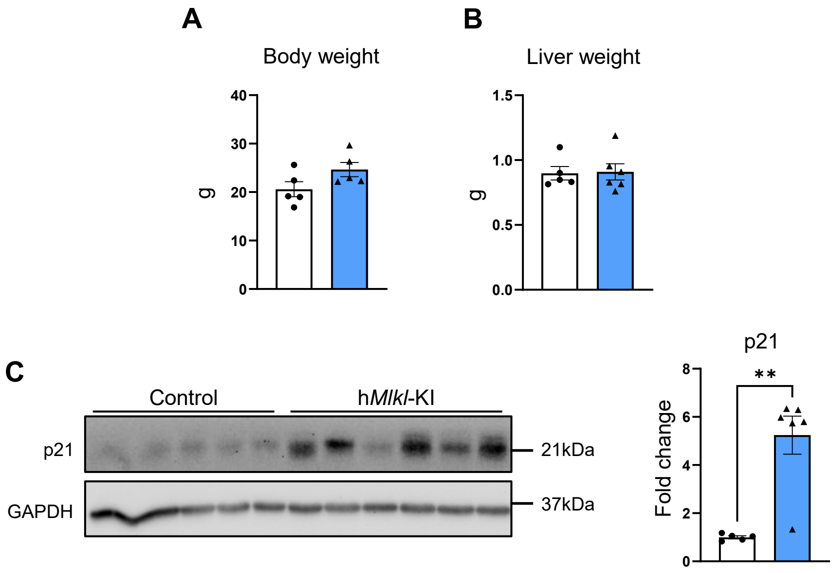

Supplementary Figure S1. Characterization of 6-month-old control and h*Mlkl*-KI mice.

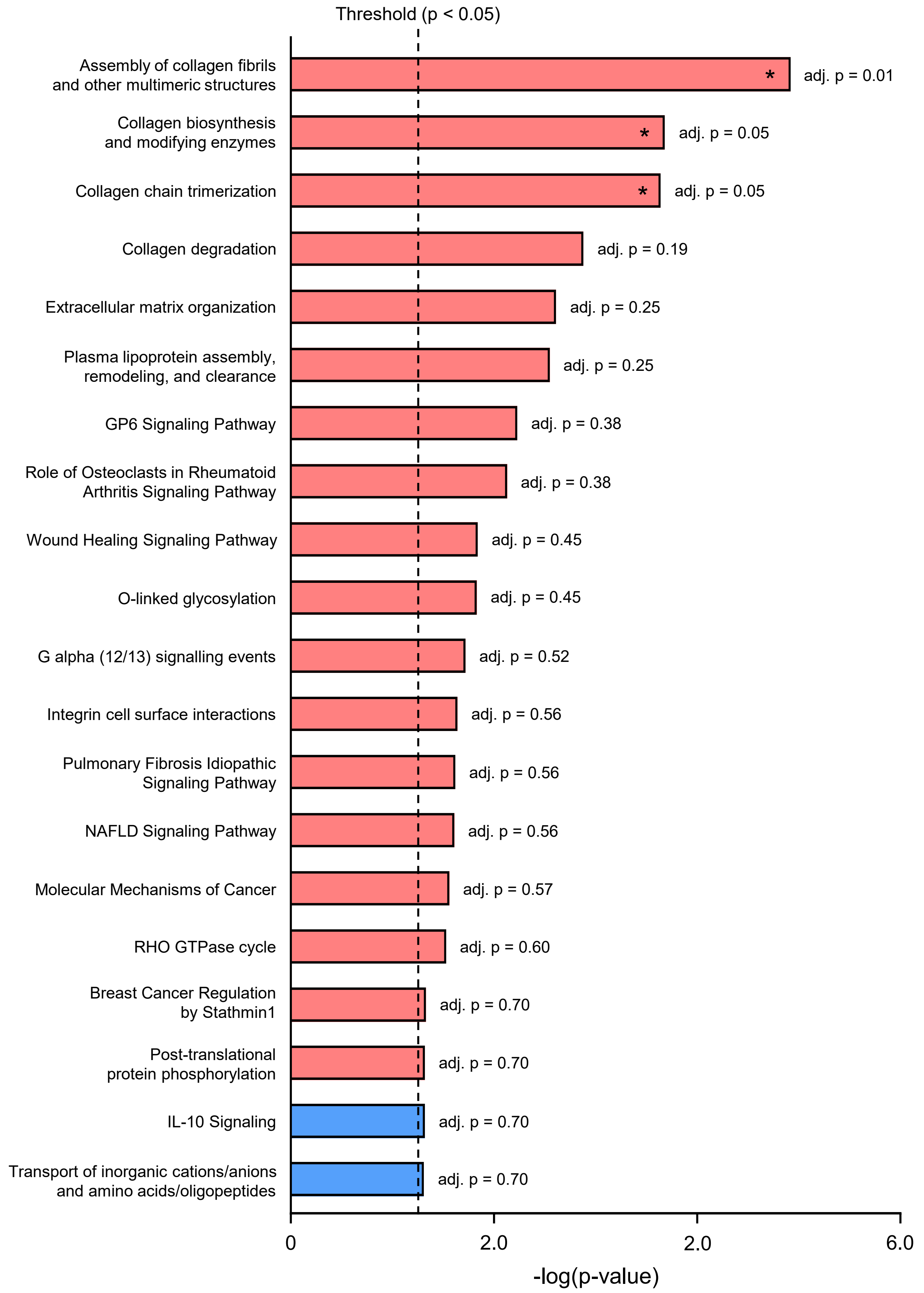

Supplementary Figure S2. Biological pathways in liver altered by MLKL overexpression.

GOBP_REPLICATIVE
_SENESCENCE

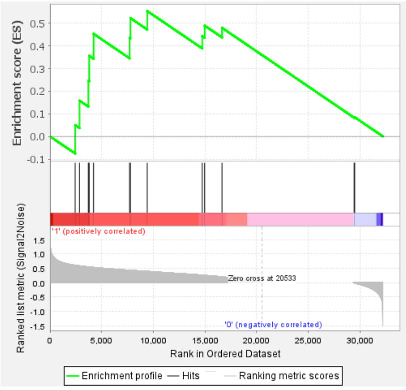

NES = 1.1632
Nominal p-value = 0.1303

REACTOME_ONCOGENE
_INDUCED_SENESCENCE

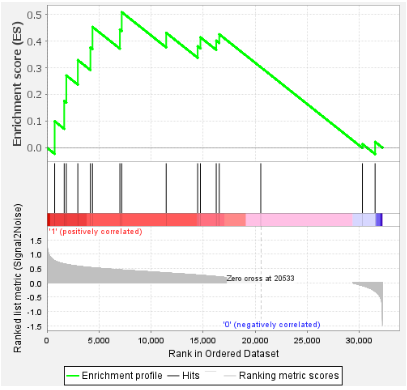

NES = 1.1015
Nominal p-value = 0.3039

KAMMINGA_SENESCENCE

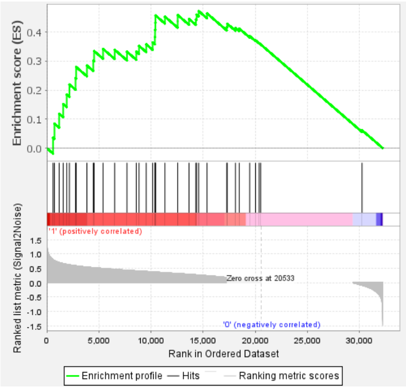

NES = 1.0923
Nominal p-value = 0.2396

REACTOME_CELLULAR
_SENESCENCE

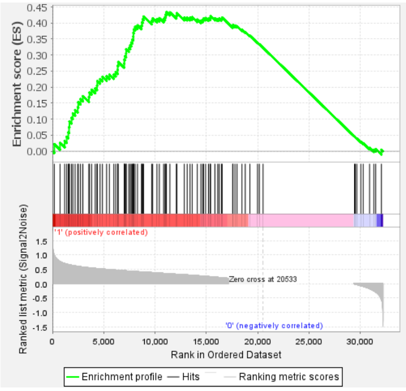

NES = 1.0389
Nominal p-value = 0.5347

REACTOME_OXIDATIVE
_STRESS_INDUCED
_SENESCENCE

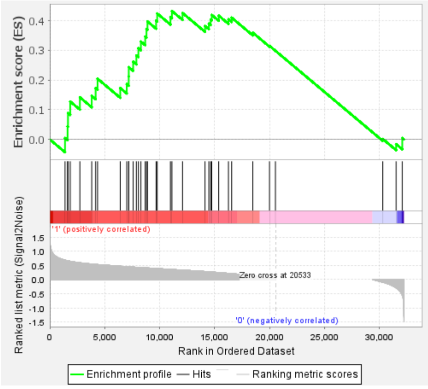

NES = 1.0371
Nominal p-value = 0.5505

SAUL_SEN_MAYO

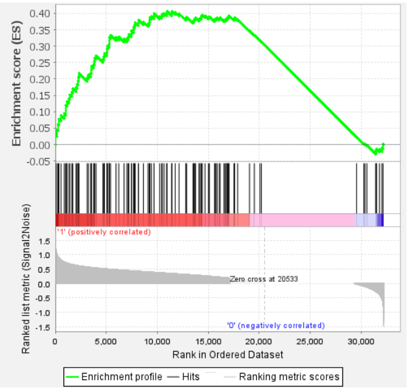

NES = 1.0087
Nominal p-value = 0.4702

REACTOME_DNA-DAMAGE
_TELOMERE_STRESS
_INDUCED_SENESCENCE

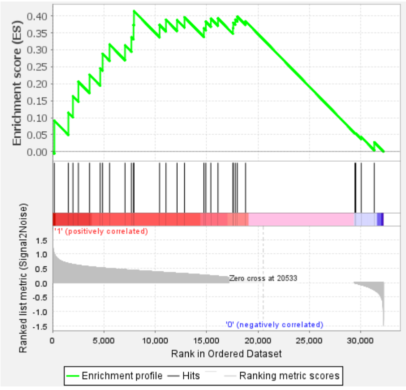

NES = 0.9480
Nominal p-value = 0.6713

Supplementary Figure S3. GSEA enrichment plots are presented for various cellular senescence gene sets.

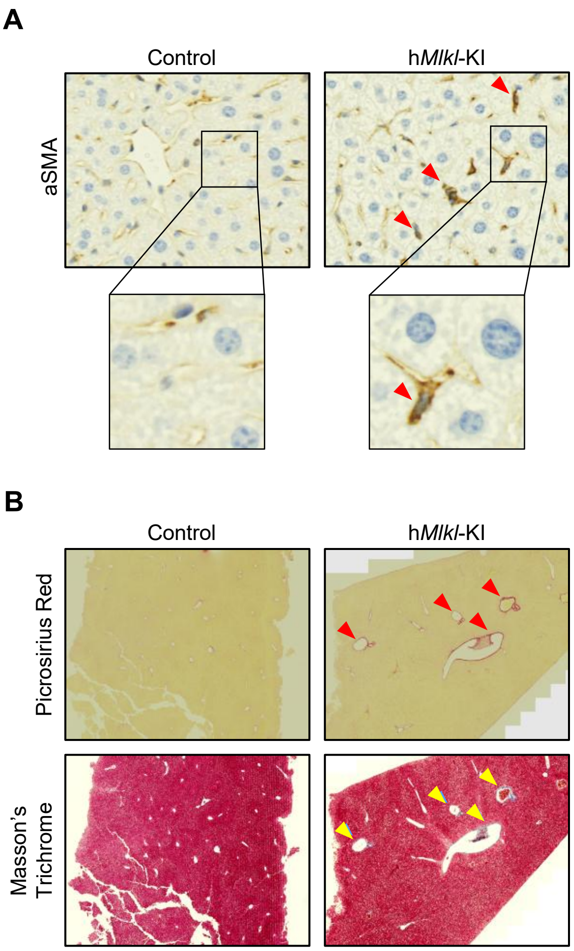

Supplementary Figure S4. Representative images of liver tissue from 6-month-old control and h*Mlkl*-KI mice stained for αSMA or collagen.

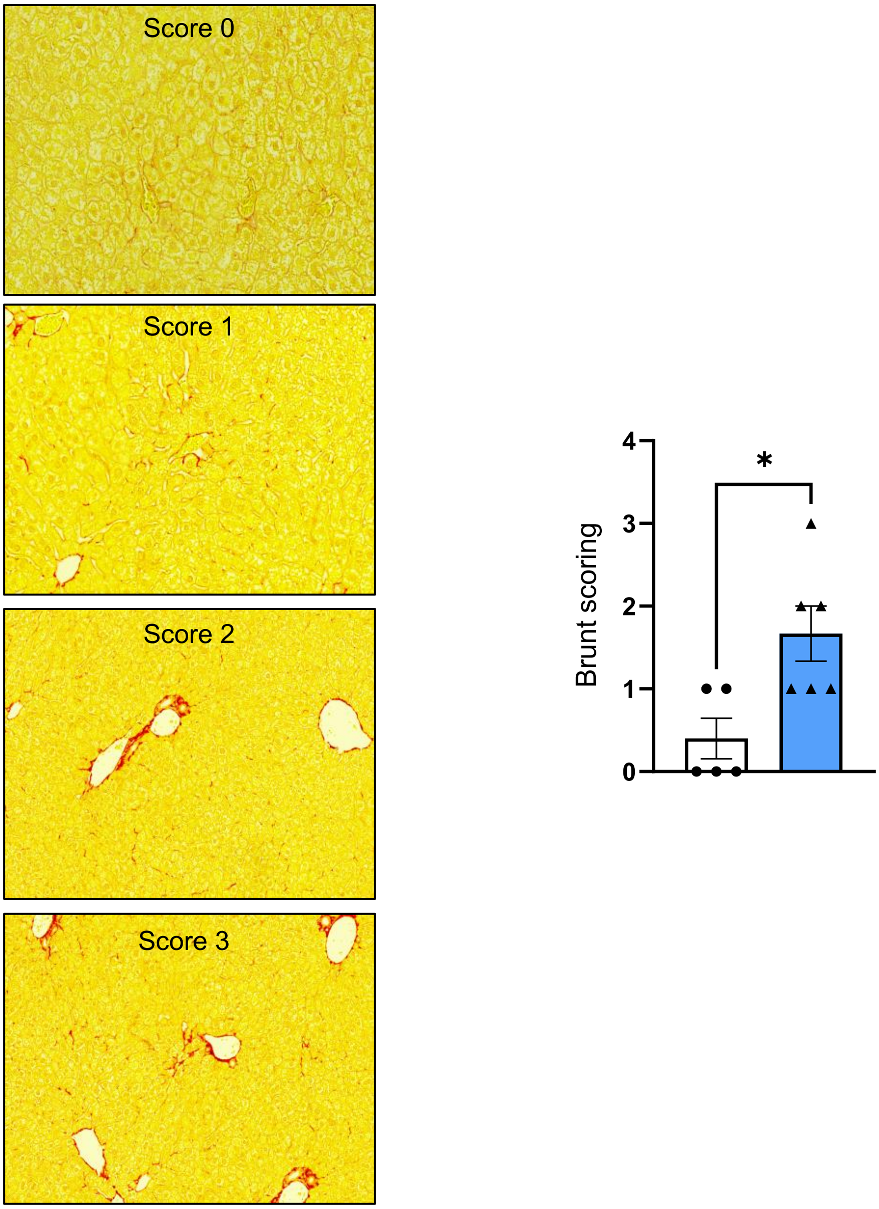

Supplementary Figure S5. The Brunt score for fibrosis is increased in the livers of h*Mlkl*-KI mice compared to control mice.
