# Supplementary material for: Necroptosis induced by MLKL overexpression in liver triggers cellular senescence and leads to chronic inflammation and fibrosis
Source: GeroScience. 2025 Nov 24;48(2):1917–35. doi: 10.1007/s11357-025-01994-y (PMC12972278; doi:10.1007/s11357-025-01994-y)
Supplement: Supplementary file 2 — (DOCX 29.3 KB) [file 11357_2025_1994_MOESM2_ESM.docx]

**Supplementary Figure S1. Characterization of 6-month-old control and h*Mlkl*-KI mice.** Body (**Panel A**) and the liver weights (**Panel B**). **Panel C**: Western blot of p21 with a graph quantifying the levels of p21. Data presented in the graphs are for individual control (white bars) and h*Mlkl*-KI (blue bars) mice with the mean ± SEM for 5 to 6 mice per group. Statistical significance was assessed by two-tailed Student’s unpaired t-tests (body and liver weights) and Mann-Whitney U test (p21). **p < 0.01.

**Supplementary Figure S2. Biological pathways in liver altered by MLKL overexpression.** The 420 DEGs were subjected to IPA, and the 20 pathways that were above the threshold (p < 0.05) before post-hoc analysis and the adj. p-values for each pathway after post-hoc analysis. Blue bars represent downregulated pathways and red bars represent upregulated pathways in the livers of h*Mlkl*-KI mice compared to control mice.

**Supplementary Figure S3. GSEA enrichment plots are presented for seven different cellular senescence gene sets.**

**Supplementary Figure S4. Representative images of liver tissue from 6-month-old control and hMlkl-KI mice stained for αSMA or collagen. Panel A:** Images of αSMA staining of sections of liver tissue from control and h*Mlkl*-KI mice (20x magnification) with arrows indicating αSMA-positive cells (i.e., activated myofibroblasts). The αSMA-positive cells were identified by their characteristic elongated or spindle-shaped morphology with the αSMA-positive staining localized in the cytoplasm and surrounding the blue-stained nuclei (shown by the arrow) in the enlarged insert. **Panel B:** Images of PSR or MT staining with arrows showing the collagen deposits in the liver tissue from control and h*Mlkl*-KI mice (1x magnification).

**Supplementary Figure S5. The Brunt score for fibrosis is increased in the livers of h*Mlkl*-KI mice compared to control mice**. Representative images (20x magnification) of PSR-stained slides from the livers of 6-month-old mice in the study are shown for the following Brunt scores: 0 (none) from a control mouse and 1 (mild perisinusoidal fibrosis), 2 (zone 3 periportal fibrosis), and 3 (bridging fibrosis) each from three different h*Mlkl*-KI mice. The graph shows data from control (white bars) and h*Mlkl*-KI (blue bars) mice presented as the mean ± SEM for 5 to 6 mice per group. Statistical significance was assessed by the Mann-Whitney U test. *p < 0.05.
