# Supplementary material for: Necroptosis induced by MLKL overexpression in liver triggers cellular senescence and leads to chronic inflammation and fibrosis
Source: GeroScience. 2025 Nov 24;48(2):1917–35. doi: 10.1007/s11357-025-01994-y (PMC12972278; doi:10.1007/s11357-025-01994-y)
Supplement: Supplementary file 3 — (DOCX 18.1 KB) [file 11357_2025_1994_MOESM3_ESM.docx]

**Supplementary Table 1.** List of RT-qPCR primers used to measure the transcript levels of corresponding genes.

| **Transcript** | **Forward Sequence** | **Reverse Sequence** |
| --- | --- | --- |
| TNFα | 5’-CACAGAAAGCATGATCCGCGACGT-3’ | 5’-CGGCAGAGAGGAGGTTGACTTTCT-3’ |
| IL-6 | 5’-TGGTACTCCAGAAGACCAGAGG-3’ | 5’-AACGATGATGCACTTGCAGA-3’ |
| IL-1β | 5’-AGGTCAAAGGTTTGGAAGCA-3’ | 5’- TGAAGCAGCTATGGCAACTG -3’ |
| F4/80 | 5'-CCCCAGTGTCCTTACAGAGTG-3' | 5'-GTGCCCAGAGTGGATGTCT-3' |
| CD68 | 5’-CCACAGGCAGCACAGTGGAC-3’ | 5’-TCCACAGCAGAAGCTTTGGCCC-3’ |
| CD206 | 5’-ACTACACACTCATCCATTACAACCAA-3’ | 5'-GGCACCTATCACAATCAGGAGGA-3' |
| TGFβ | 5’-ACCATGCCAACTTCTGTCTGGGAC-3’ | 5’-ACAACTGCTCCACCTTGGGCTTG-3’ |
| Col1α1 | 5’-GCTCCTCTTAGGGGCCACT-3’ | 5’-CCACGTCTCACCATTGGGG-3’ |
| Col3α1 | 5’-CTGTAACATGGAAACTGGGGAAA-3’ | 5’-CCATAGCTGAACTGAAAACCACC-3’ |
| p16 | 5'-CCCAACGCCCCGAACT-3’ | 5'-GCAGAAGAGCTGCTACGTGAA-3’ |
| p21 | 5’-GGCAGACCAGCCTGACAGAT-3' | 5’-TTCAGGGTTTTCTCTTGCAGAAG-3' |
| p53 | 5’-GTATTTCACCCTCAAGATCC-3' | 5’-TGGGCATCCTTTAACTCTA-3' |
| PAI-1 | 5′-GACACCCTCAGCATGTTCATC-3′ | 5′-AGGGTTGCACTAAACATGTCAG-3′ |
| CCL2 | 5′-TTAAAAACCTGGATCGGAACCAA-3′ | 5′-GCATTAGCTTCAGATTTACGGGT-3′ |
| IFNγ | 5’-ATGAACGCTACACACTGCATC-3’ | 5’CCATCCTTTTGCCAGTTCCTC-3’ |
| CXCL-1 | 5’-ACCCGCTCGCTTCTCTGT-3’ | 5’- AAGGGAGCTTCAGGGTCAAG-3’ |
| CXCL-8 | 5’-AGACAGCAGAGCACACAAGC-3’ | 5’-ATGGTTCCTTCCGGTGGT-3’ |
| CXCL-10 | 5’-CCAAGTGCTGCCGTCATTTTC-3’ | 5’-GGCTCGCAGGGATGATTTCAA-3' |
| MMP-9 | 5′-CTGGACAGCCAGACACTAAAG-3′ | 5′-CTCGCGGCAAGTCTTCAGAG-3′ |
| MMP-12 | 5’-TGCACTCTGCTGAAAGGAGTCT-3’ | 5’-GTCATTGGAATTCTGTCCTTTCCA-3’ |
| p19 | 5'-GGGTCGCAGGTTCTTGGTC-3' | 5'-AATCTGCACCGTAGTTGAGCA-3' |
| GDF-15 | 5′-GTTAGCCAAAGACTGCCACTG-3′ | 5′-CCTTGAGCCCATTCCACA-3′ |
| p53 | 5'-GTATTTCACCCTCAAGATCC-3' | 5'-TGGGCATCCTTTAACTCTA-3' |
| β-microglobulin | 5′-CACTGACCGGCCTGTATGC-3′ | 5′-GGGTGGCGTGAGTATACTTGAAT-3′ |

**Supplementary Table 2.** List of 420 DEGs in the livers of the h*Mlkl*-KI mice compared to the control mice.
